# CT-Guided Pelvic Lymph Nodal Brachytherapy

**DOI:** 10.3389/fonc.2020.532555

**Published:** 2021-02-19

**Authors:** Hiroaki Kunogi, I-Chow Hsu, Nanae Yamaguchi, Soshi Kusunoki, Keiko Nakagawa, Yayoi Sugimori, Kazunari Fujino, Yasuhisa Terao, Daiki Ogishima, Ryoichi Yoshimura, Keisuke Sasai

**Affiliations:** ^1^Department of Radiation Oncology, Juntendo University, Tokyo, Japan; ^2^Department of Radiation Oncology, University of California San Francisco, San Francisco, CA, United States; ^3^Department of Gynecology, Juntendo University, Tokyo, Japan; ^4^Department of Radiation Oncology, Tokyo Medical Dental University, Tokyo, Japan

**Keywords:** CT guidance, gynecological malignancies, interstitial brachytherapy, nodal brachytherapy, pelvic lymph node

## Abstract

**Purpose:**

This is a report of our initial experience using computed tomography (CT)-guided interstitial high dose rate (HDR) brachytherapy to treat bulky pelvic nodal metastases as a part of definitive radiotherapy.

**Material and methods:**

Between February 2015 and April 2019, 14 cervical/endometrial cancer patients presenting with bulky pelvic node(s) underwent nodal interstitial brachytherapy boost in our institution. In total, 17 nodes were treated. The median maximum diameters of the positive nodes at the time of diagnosis and at the first nodal implant were 25 mm (range: 10–65 mm) and 16 mm (range: 9–51 mm), respectively. Dosimetry data of the lymph nodal target volume and small bowel were collected and compared using the paired-sample *t-*test. Treatment-related toxicities were classified using the Common Terminology Criteria for Adverse Events version 4.0.

**Results:**

The median follow-up time for all patients was 26 months. Local recurrence in pelvic nodes occurred in one patient (7%) after 16 months. One patient experienced grade 3 bladder bleeding, and one patient experienced grade 2 pubic bone fracture. No patient had grade 2 or greater gastrointestinal toxicity. In the dosimetric analysis, the mean nodal brachytherapy D_90%_ in terms of the total equivalent dose of 2 Gy (EQD2) was 65.6 Gyαβ10. The mean small bowel dose (SBD)_0.1cc_ and SBD_1cc_ in terms of the total EQD2 were 60.4 and 56.5 Gyαβ3, respectively. Nodal D_90%_ was significantly higher in terms of the total EQD2 than the SBD_0.1cc_ (*p* = 0.003) and SBD_1cc_ (*p* < 0.001). The Kaplan-Meier 2-year pelvic control estimate was 90%.

**Conclusions:**

CT-guided interstitial HDR pelvic nodal brachytherapy appears to be well tolerated with excellent local control in cervical or endometrial cancer patients with bulky pelvic nodes. This approach may offer a useful therapeutic option for unresected bulky pelvic nodes.

## Introduction

Brachytherapy offers both precise dose delivery and highly conformal dose distribution. By using brachytherapy combined with external beam radiotherapy (EBRT), even large cervical/endometrial cancer can be cured (1–3). Advanced gynecological cancer often presents with enlarged pelvic nodal metastases. For definitive therapy, local control of both primary and metastatic nodal disease is required. Similarly, in post-operative patients with pelvic nodal recurrence, an adequate dose must be delivered to achieve local control (4, 5).

It is often difficult to deliver an adequate dose to large pelvic nodal metastases due to their proximity to the small bowel, the dose-limiting structure. The dose to the pelvic nodes cannot be safely escalated beyond 55 Gy because of small bowel tolerance. NCCN guidelines (4) state that highly conformal boosts of an additional 10–15 Gy (approximate total dose of 55–60 Gy) may be considered for limited volumes of gross unresected nodal disease.

Interstitial brachytherapy (ISBT) can increase the dose to pelvic nodes beyond the level achievable by EBRT. A larger curative effect may be expected from adding ISBT while maintaining dose constraints to organs at risk (OARs). Dose escalation using brachytherapy for large lymph nodes or nodes adjacent to the bowel can be part of definitive radiotherapy. Indeed, previous studies have reported successful clinical outcomes using nodal brachytherapy. Yao et al. (6) reported on the feasibility, efficacy and safety of 17 patients re-irradiated with computed tomography (CT)-guided ^125^I permanent seed implant of the retroperitoneal nodes. Kishi et al. (7) reported a case re-irradiation of paraaortic node using CT-guided high-dose-rate (HDR) ISBT and had no evidence of recurrence at 13 months after ISBT. Yoshida et al. (8) reported a case of trans-rectal ultrasound (TRUS)-guided HDR ISBT of an internal iliac node that developed marginal recurrence at 15 months after complete response due to ISBT. As bowel morbidity is a major side effect of dose escalation (9), our group performed HDR ISBT for pelvic nodes to achieve higher dose delivery while maintaining the standard small bowel dose constraint.

This novel ISBT technique can be a used as boost after pelvic radiotherapy or stand-alone brachytherapy monotherapy. The purpose of this study was to investigate the clinical application of this technique for pelvic nodal metastases from gynecologic cancer with special attention to treatment safety.

## Materials and Methods

### Patients and Indications for Nodal ISBT

The clinical data of 14 patients treated with HDR ISBT for pelvic nodes between February 2015 and April 2019 at our institution were retrospectively reviewed. All 14 patients underwent HDR ISBT using microSelectron- HDR^®^ (Nucletron B.V., Veenendaal, The Netherlands) with an ^192^Ir source. **Table 1** shows the patient characteristics. Eligibility criteria for nodal ISBT were as follows: 1) pelvic node metastases were diagnosed using positron emission tomography with ^18^F-labeled fluoro-2-deoxyglucose/computed tomography (^18^F-FDG-PET/CT) or CT (10), 2) the maximum number of positive nodes was limited to two and the maximum diameter of at least one of the positive nodes was “bulky” (>15 mm) at the time of diagnosis, and 3) positive nodes could be implanted using brachytherapy needle(s). Patient’s eligibility was decided based on if the implant catheter(s) can be inserted into the target to allow adequate coverage of the target without puncturing other organs at risk. All the evaluations were performed by one brachytherapist (HK). All lower pelvic nodes can be reached with this technique; however, cases with disease at common iliac node or superior were excluded because the brachytherapy needle cannot reach these nodes. Prior to treatment, written informed consent for EBRT and ISBT, including nodal ISBT, as a component of definitive radiotherapy was obtained. Each patient also consented to our use of their clinical data for clinical research. Patient was given the option to opt out any portion of the treatment. This retrospective study was approved by the ethics committee of our institution (approval no. 17-291).

**Table 1:** Patient characteristics.

Patient characteristics	
Age (years)	
Median (range)	61 (40–89)
Primary	
Cervix	11 (79%)
Uterine corpus	2 (14%)
Unknown	1 (7%)
Histology	
SqCC	8 (57%)
ADCA	4 (29%)
Adeno-SqCC	1 (7%)
Mixed	1 (7%)
Presentation	
Initial treatment	10 (71%)
Vaginal recurrence (post hysterectomy)	2 (14%)
Node recurrence only (post hysterectomy)	2 (14%)
Number of nodes at implant	
Median (range)	1 (1–2)
Diameter of node at diagnosis (mm)	
Median (range)	25 (10–65)
Diameter of node at implant (mm)	
Median (range)	16 (9–51)

ADCA, Adenocarcinoma; SqCC, Squamous cell carcinoma.

### External Beam Radiotherapy

Whole-pelvis EBRT (50.4 Gy in 28 fractions) using 1.8 Gy per fraction was performed as initial definitive therapy or definitive treatment of recurrent disease within 2 years after hysterectomy. For recurrent cases more than 2 years after hysterectomy, local EBRT (50 Gy in 25 fractions) was delivered to the nodal disease plus a margin. The recurrence in these cases were assumed to be confined to the node only. Extended field radiotherapy was used in cases in which the nodal disease extended beyond the common iliac nodes. A total dose of 50.4 Gy in 28 fractions was delivered to pelvic and para-aortic nodes.

For whole-pelvis EBRT planning, the clinical target volume (CTV) was contoured, including both the primary CTV and pelvic nodal CTV. The pelvic nodes were delineated in accordance with the Japan Clinical Oncology Group Gynecologic Cancer Study Group (JCOG-GCSG) consensus guidelines for pelvic node delineation (11). For extended-field intensity-modulated radiotherapy (EF-IMRT) planning, the CTV was contoured, including the primary CTV, pelvic nodal CTV, and para-aortic lymph nodes according to our previously reported procedure (12). Briefly, para-aortic nodes included those in the region between the psoas muscles, superiorly to the top of the renal arteries, and anteriorly encompassing the aorta and inferior vena cava with a 7-mm margin. The CTV was isotropically expanded by 5–7 mm to create the planning target volume (PTV). Whole-pelvis EBRT or extended-field radiotherapy plans were generated using box field or IMRT without a simultaneous integrated boost (SIB) technique. The type of EBRT used at our institution changed over the course of the study. Box field plans were generated using gantry angles of 0°, 90°, 180°, and 270° with the field-in-field technique. IMRT plans were generated using seven fixed-fields on the Eclipse Planning System (version 11.0; Varian Medical Systems, Palo Alto, CA, USA). All patients were treated with 10-MV photons. IMRT plans were optimized using the “Normal Tissue Objective” function in the Eclipse Planning System to spare the OARs (small bowel, bladder, and rectum). A plan was accepted if 95% of the PTV volume received 98% of the prescribed dose, with the maximum dose being <110%, and 98% of the PTV receiving 95% of the prescribed dose, while keeping the irradiated volumes of small bowel, bladder and rectum as low as possible.

### Nodal Brachytherapy Procedure

**Figure 1:**
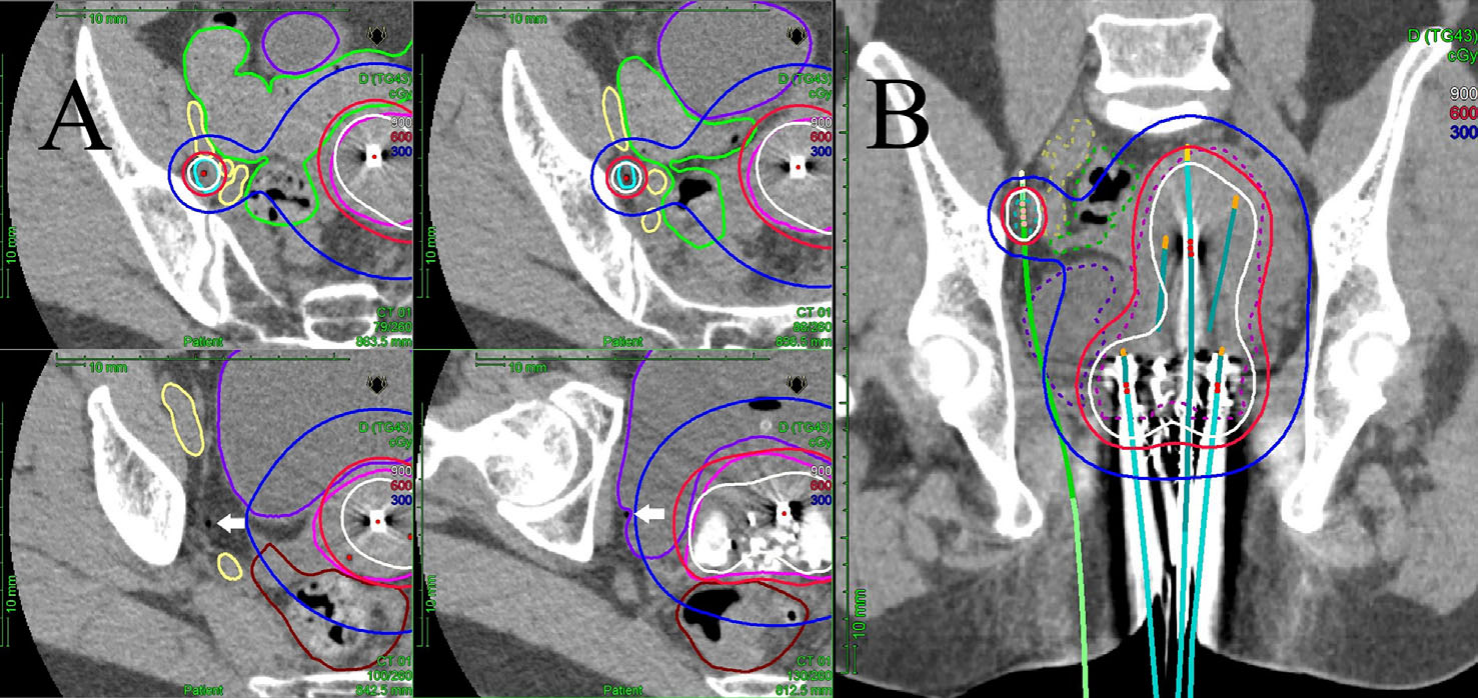
**(A)** Multi-slice axial CT images of the right obturator node (cyan line), uterine target (pink line), bowel (green line), external/internal iliac vessel (yellow line), rectum (brown line), and bladder (purple line) receiving 50% (blue lines), 100% (red lines), and 150% (white lines) of the prescription dose (6 Gy) in a representative patient with a right obturator node metastasis. One interstitial flexible catheter (white arrows) was inserted in the lithotomy position passing from the inside of the pubic bone through the outside of the bladder up to the front of the right sacroiliac joint using TRUS and CT guidance in the HDR room with dedicated CT, while avoiding bladder and external/internal iliac vessels. TRUS is commonly used for implantation from the perineum, and CT is used to adjust catheter positions at a deeper level. An iterative approach of catheter adjustment and CT image acquisition allows precise placement of the catheter in the target volume. **(B)** A coronal CT image of the uterine target (pink dashed line), the right obturator node (cyan dashed line), bowel (green dashed line), external/internal iliac vessel (yellow dashed line), rectum (brown line), and bladder (purple dashed line) receiving 50% (blue lines), 100% (red lines), and 150% (white lines) of the prescription dose (6 Gy) from interstitial and intracavitary applicators in a representative patient with a right obturator node metastasis. It is apparent that one interstitial flexible catheter (light green) is implanted, passing from the inside of the pubic bone through the outside of the bladder, so that the catheter can be inserted to the central part of the node.

**Figure 2:**
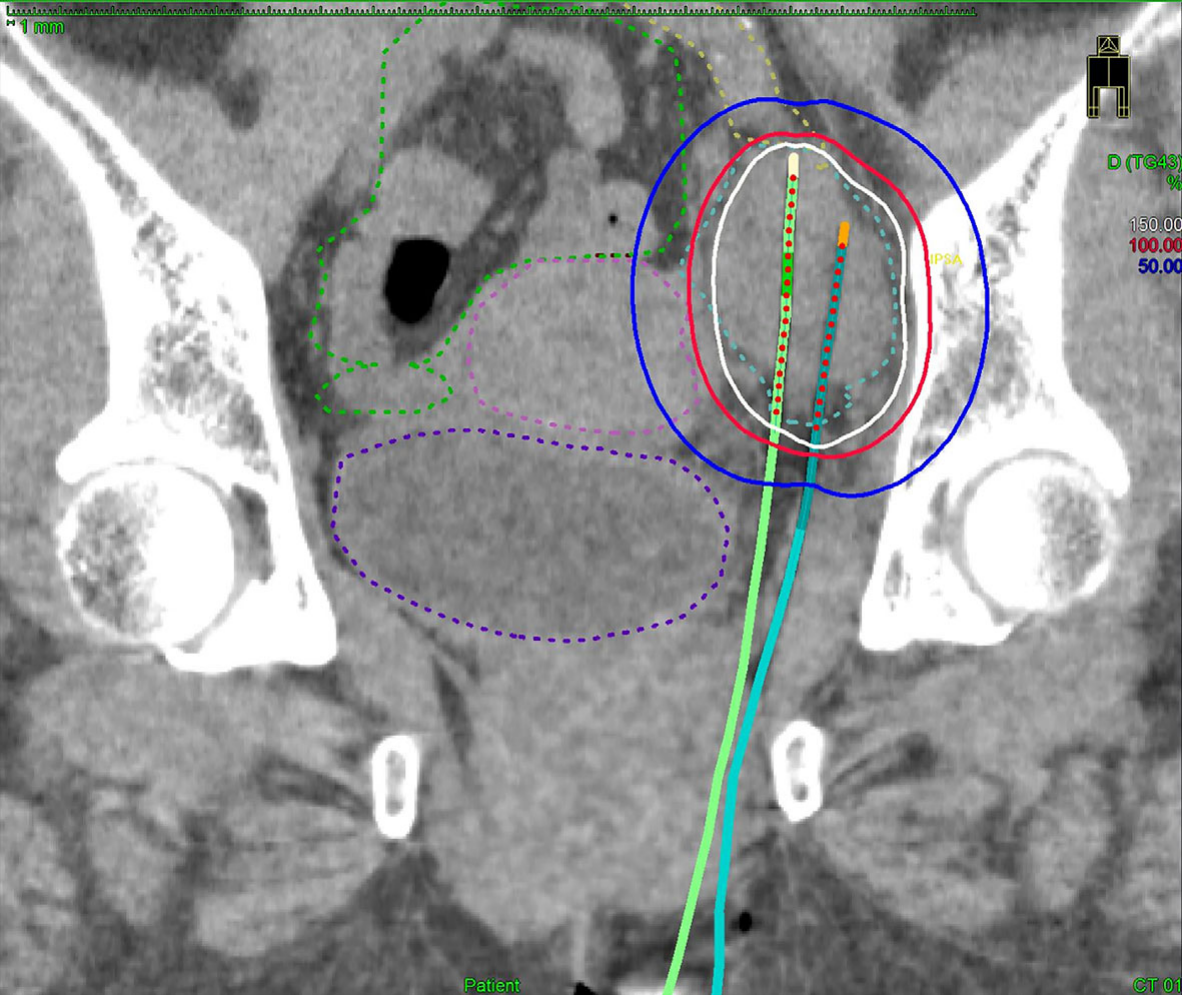
A coronal CT image of the bulkiest obturator node (blue dashed line), uterus (pink dashed line), bowel (green dashed line), external iliac vessel (yellow dashed line), bladder (purple dashed line) receiving 50% (blue lines), 100% (red lines), and 150% (white lines) of the prescription dose (6 Gy) from two implanted needles.

**Figure 3:**
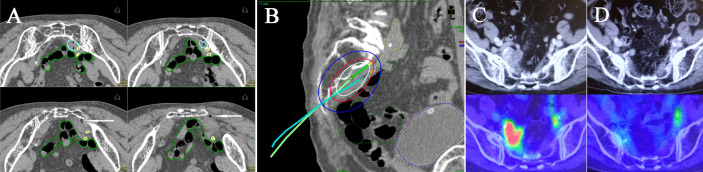
Multi-slice axial CT images **(A)** of implanted needles and a sagittal CT image **(B)** of the right internal iliac node (cyan dashed line), bowel (green dashed line), common/internal iliac vessels (yellow dashed line), and bladder (purple dashed line) receiving 50% (blue lines), 100% (red lines), and 150% (white lines) of the prescription dose (6 Gy) in a representative post-operative patient. Pre-treatment CT/^18^F-FDG-PET/CT images **(C)** and post-treatment CT/^18^F-FDG-PET/CT images **(D)**. Both insertion points and directions were decided using pre-treatment CT. Two flexible interstitial needles (white arrows) were obliquely implanted in the prone position, passing from under the right sacroiliac joint through the front of the piriformis muscle up to the front of the second sacral vertebrae under CT guidance (no contrast medium) in the HDR room with dedicated CT. Implantation was performed under local anesthesia alone, with care taken not to damage the bowel or common/internal iliac vessels. The catheter was inserted into the central part of the node using an iterative approach of catheter implantation and adjustment with serial CT image acquisition. MPR CT was used to reconstruct the axial images into oblique anatomical planes. The patient was irradiated in the prone position in the HDR room with dedicated CT.

Brachytherapy was performed under intravenous and/or local anesthesia. In patients with uterine cancer or recurrence in vaginal cuff, three or four fractions of 6 Gy were delivered to the primary site at one fraction per week. For brachytherapy of the primary site, we used either an unshielded Fletcher Williamson HDR applicator or a cylinder applicator, such as the MUPIT applicator, in combination with either metallic trocar point needles or flexible needles (ProGuide Sharp Needle^®^; Nucletron B.V., Veenendaal, The Netherlands). The primary and nodal brachytherapy were performed together when possible. Using TRUS and CT guidance, one or two needles (metallic or flexible) for each node were implanted using the free hand technique (**Figures 1**–**3**). To change the direction of the needle in the deep parts of the pelvis, larger diameter flexible needles, such as 6 Fr, were preferred because of its stiffness. Before implantation, both the insertion points and needle paths were determined using CT. An iterative approach of catheter implantation and adjustment followed by serial CT imaging was used. No intravenous CT contrast was used to visualize vessels near the lymph target. The border between the vessels and targeted nodes was distinguished using thin-slice CT guidance with 1–3 mm spacing. The needles were carefully and slowly advanced under CT guidance, while avoiding blood vessel, nerve, bowel, and bladder. The first three patients were implanted on the CT table. After the implant and planning-CT scan, these three patients were moved to the HDR room and treated. Subsequent 10 patients were implanted in the HDR room with a dedicated CT system capable of scanning patients in the lithotomy position. Most patients were treated in the lithotomy position on the CT table without moving after implantation. One patient (**Figure 3**) was implanted and treated in a prone position in the HDR room with dedicated CT. The prone position was chosen based on the pre-treatment CT. It was easier to approach the nodal target volume from the perineum while avoiding the bowel. Multi-planar reconstruction (MPR) CT, a post-processing technique, was used to reconstruct the axial images into oblique anatomical planes to assist with the image guided process. All patients received a single fraction on the day of the implant, after which the implant was removed.

### CT-Based Planning for Nodal HDR Brachytherapy

For brachytherapy planning, median CT spacing was 1 mm (1–2.5 mm). The nodal planning target volume (PTVn) included the gross node on the axial CT slices without any expansion margins. OAR volumes included the small bowel (13). Occasionally, the sigmoid colon was included in the small bowel volume. This was done so the stricter OAR constraint (small bowel) was used during dose calculation. The planning organ at risk volume (PRV) was contoured as an OAR (no margin was added to the contoured OAR).

Three-dimensional brachytherapy plans were created using the inverse planning simulated annealing (IPSA) algorithm or a graphical optimization tool in the Elekta Nucletron Oncentra Planning System (version 4.5). The prescription dose was set to 6 Gy per fraction. The plan was generated according to the following dose requirements: PTVn V_100%_ value (volume receiving 100% of the prescribed dose) is >90%, and small bowel D_1cc_ value (SBD_1cc_; i.e., the minimum dose in the maximum irradiated 1 cc of the small bowel) is < 85% of the reference prescription dose (6 Gy). The planned goal was to provide the following small bowel constraint: SBD_1cc_: < 10 Gy [equivalent dose in 2 Gy (EQD2)]; total dose, <60 Gy. Based on the small bowel constraint, we determined the fraction number for nodal brachytherapy. If target volume reduction is still insufficient at the time of the third nodal brachytherapy, a maximum SBD_1cc_ of 65 Gy (EQD2, in total) was accepted.

### Evaluation of Dosimetric Parameters of the Nodal Target Versus Small Bowel

The metrics used were: D_90%_ and D_95%_ (i.e., the minimum dose delivered to 90% and 95% of the volume of the PTVn, respectively), SBD_0.1cc_ and SBD_1cc_ (i.e., the minimum dose in the maximum irradiated 0.1 cc and 1 cc of the small bowel, respectively). When brachytherapy of the primary tumor and bulky node(s) was performed together, only the bowel dose near the nodal implant was included in the dosimetry analysis. Values are expressed in EQD2, assuming α/β of 10 for the PTVn and 3 for the OARs.

### Patient Follow-Up (Lymph Node Control and Toxicity)

After treatment, patients were followed without adjuvant therapy every 1–2 months for 2 years and every 2 or 3 months thereafter. The examination consisted of a physical examination, routine blood cell counts, chemistry profile, a blood tumor marker test, and transvaginal/transrectal ultrasound. In principle, CT or ^18^F-FDG-PET/CT was performed every 6 months for 2 years and every 12 months thereafter. Pelvic MRI was also performed every 6 months for 2 years and every 12 months thereafter. Toxicities were evaluated using the Common Terminology Criteria for Adverse Events version 4.0 (CTCAE-4) (14).

### Statistics

The dosimetry data of the lymph nodal target volume and small bowel were compared using the paired-sample *t-*test. Statistical analyses were performed using SPSS ver. 18 (SPSS Inc., Chicago, IL, USA). *P-*values less than 0.05 were considered statistically significant.

## Results

### Patient Characteristics

The median age was 61 years, ranging from 40 to 89 years. In total, 17 nodes, including 16 obturator nodes and an internal iliac node, were implanted in the 14 patients, and the median maximum diameters of the positive nodes at the time of diagnosis and the first nodal implant were 25 mm (range: 10–65 mm) and 16 mm (range: 9–51 mm), respectively. In thirteen patients (93%), pelvic node metastases were diagnosed using ^18^F-FDG-PET/CT. In the remaining one patient, pelvic node metastases were diagnosed using CT and pelvic MRI. The median number of nodal implant needles per node was 1 (range: 1–2). One patient with an unknown primary (Patient #4 in **Table 2**) had a bulky obturator node (squamous cell carcinoma, maximum diameter: 65 mm). This patient had been clinically suspected to be a cervical cancer patient based on age, sex, histology, and the position of the node, and was treated accordingly.

**Table 2:** Patients/treatment characteristics and late toxicities.

Pt. No.	Node No.	EBRT(Gy)	Nodal BT (Gy/Fx)	D_90%_ in Total EQD2 (Gy)	D_95%_ in Total EQD2 (Gy)	SBD_0.1cc_ in Total EQD2 (Gy)	SBD_1cc_ in Total EQD2 (Gy)	Pelvic Control	Grade 2–4 toxicity	F/U (month)
1	1	50.4	6Gy/1Fx	54.9	54.3	54.2	51.8	CR	None	56
	2		6Gy/1Fx	54.7	54.1	61.1	55.6	CR		
2	3	50	12Gy/2Fx	71.7	68.3	65.2	58.3	CR	None	54
3	4	50.4	6Gy/1Fx	61.4	59.7	52	51.2	CR	Gr3 bladder bleeding	42
4	5	50.4	18Gy/3Fx	81	76.1	66.8	61.9	PR	None	31
5	6	50.4	18Gy/3Fx	75.8	72.6	71.2	65.5	PD	None	29
6	7	50.4	6Gy/1Fx	60.1	58.9	53	51.8	CR	None	9
	8		18Gy/3Fx	67.1	64.7	61	57.4	CR		
7	9	50.4	6Gy/1Fx	58.9	58	54.2	52.4	CR	Gr2 pubic bone fracture	27
8	10	50.4	6Gy/1Fx	64.6	62.7	51	50.1	CR	None	26
9	11	50.4	6Gy/1Fx	67	64.6	54.2	52.4	CR	None	26
	12		6Gy/1Fx	60.3	58.5	57.8	54	CR		
10	13	50	6Gy/1Fx	60.7	58.4	68.4	60.8	CR	None	9
11	14	50.4	12Gy/2Fx	66.6	65.6	59.4	55.3	CR	None	20
12	15	50.4	18Gy/3Fx	73.6	70.2	74.2	65.2	CR	None	17
13	16	50.4	12Gy/2Fx	67.9	66.7	62.5	58.1	CR	None	13
14	17	50.4	12Gy/2Fx	69.7	65.7	60.9	58.1	CR	None	7

BT, Brachytherapy; CR, Complete response; EBRT, external beam radiotherapy; EQD2, Equivalent dose of 2 Gy; F/U, Follow-up; PR, Partial response; PD, Progression of disease; D90% and D95%, minimum dose delivered to 90% and 95% of the volume of the nodal planning target volume, respectively.

Of the 14 patients, 9 underwent whole-pelvis EBRT (IMRT or four-field box) as the initial treatment. Two patients with recurrences in the vaginal cuff and pelvic nodes after hysterectomy underwent whole-pelvis EBRT. Two other patients with pelvic nodal recurrence only at either 30 or 48 months after hysterectomy underwent local EBRT to the recurrent nodal disease only. Elective radiation of the pelvic node was not used. The remaining patient with an unknown primary (who had been clinically suspected to have cervical cancer) underwent EF-IMRT because the bulky obturator node extended to the region of common iliac node. **Table 2** shows the treatment characteristics. The median follow-up after the first day of treatment was 26 months (range: 7–56 months).

### Evaluation of Dosimetric Parameters of the Nodal Target Versus the Small Bowel

The dosimetric parameters of the nodal target and small bowel are shown in **Table 2**. The mean D_90%_ value in terms of the total EQD2 for PTVn was 65.6 Gyαβ10 (range: 54.7–81 Gyαβ10). The mean SBD_0.1cc_ and SBD_1cc_ values were 60.4 Gyαβ3 (range: 51–74.2 Gyαβ3) and 56.5 Gyαβ3 (range: 50.1–65.5 Gyαβ3), respectively. In terms of the total EQD2, the D_90%_ value for PTVn was significantly higher than the SBD_0.1cc_ (*p* = 0.003) and SBD_1cc_ values (*p* < 0.001).

### Disease Control

Among the 14 patients at the median follow-up of 26 months, the Kaplan-Meier 2-year pelvic control estimate for primary and metastatic nodal disease in patients with “bulky” nodes without adjuvant therapy was 90%. No recurrence occurred at the primary site after image-guided ISBT.

One cervical cancer patient showed nodal recurrence. Sufficient reduction of the node had not been achieved at the time of the third nodal brachytherapy, so SBD_1cc_ of about 65 Gy (EQD2) was accepted. However, the obturator node increased in size, and a relapse was suspected on MRI and ^18^F-FDG-PET/CT. This case underwent local resection for the growing pelvic node within the irradiated region, and nodal recurrence was pathologically confirmed at a follow-up time of 16 months. However, local recurrences occurred again after 3 months, and this case underwent systemic chemotherapy. Another patient developed metastases to the para-aortic nodes, common iliac nodes, and nodes on both sides of the groin on ^18^F-FDG-PET/CT at 6 months follow up, although the bulky internal iliac node was controlled. This case was treated with systemic chemotherapy after failure and died due to chemotherapy induced grade 4 neutropenia and septic shock. Another patient had distant failure (both nodal metastases above the collarbone and one right lung metastasis) after 22 months. At 22 months, this case was treated using EBRT (60 Gy in 30 fractions) with concurrent chemotherapy (weekly cisplatin, 40 mg/m^2^) to nodes above the collarbone, and was treated with stereotactic body radiotherapy (SBRT, 60 Gy in 8 fractions) to the lung metastasis. This patient continued to maintain a complete remission without further therapy. The patient with bulkiest obturator node showed a partial response. This case underwent watchful waiting (the maximum diameter of the node at the last follow-up of 31 months: 21 mm) because the necrotic node continues to decrease in size on enhanced CT and transvaginal ultrasound. One patient died because of her advanced age and frailty after radiotherapy. No recurrence occurred after radiotherapy in the remaining nine patients.

### Toxicity

No patient had grade 2 or higher gastrointestinal (GI) toxicity. One patient experienced grade 1 large intestinal bleeding caused by primary or nodal brachytherapy at 11 months. One patient, whose nodal target was about 3 cm from the bladder wall, experienced grade 3 bladder bleeding requiring hospitalization at 35 months. One patient experienced grade 1 urinary frequency from 4 months to the last follow-up. One patient, whose nodal target was about 10 cm from the pubic bone, experienced a pubic bone fracture that was managed with conservative therapy (grade 2) at 16 months. No patient had toxicity related to blood vessel or ureter. One patient experienced grade 1 right leg edema at 52 months and grade 1 radiation pneumonitis caused by lung SBRT.

## Discussion

Based on our results, we believe that ISBT is a safe and effective treatment for bulky pelvic nodes and should be considered as a treatment option for eligible patients. In gynecologic oncology radiotherapy, the control rate of the primary site is improved by brachytherapy. We believe the same holds true for bulky pelvic nodes. CT-guided pelvic nodal ISBT can be performed safely. Although safety and feasibility of CT-guided HDR ISBT for oligometastatic retroperitoneal nodes from different primary tumors has been reported in the past (15), the novelty of our study is that it was carried out in a relatively uniform subset of patients using the best currently available imaging technology. The excellent outcome reported in this small retrospective study supports further investigation and development of this technique.

Brachytherapy is a powerful tool that can overcome various challenges in managing bulky disease. Some authors reported using SBRT for pelvic or paraaortic nodes in gynecological cancer patients (16, 17) or IMRT for patients with pelvic or paraaortic nodes treated beyond 55 Gy using IMRT with the SIB technique (18, 19). These are very promising techniques and should be effectively utilized. However, for larger and bulkier positive nodes and for combining with primary brachytherapy to the cervix, nodal brachytherapy may have its own merits. Brachytherapy needle applicators retain their position in relation to the tumor and therefore minimized the need for additional margins. This combined with the steep dose fall off of brachytherapy can improve organ sparing. Nodal brachytherapy can be done during brachytherapy of the primary tumor. This minimize potential error in matching dose between EBRT and brachytherapy. Brachytherapy also delivers a higher internal dose that reduces the chance of survival of radioresistant tumor cells. It is impossible to know which one of these approaches will provide the best result without a randomized trial.

NCCN guidelines (4) recommend a minimum total dose of 55–60 Gy may lead to improved nodal disease control, although the optimal radiation dose has not been established. In the setting of a gross nodal recurrence, an increased dose is likely to be required since there are more tumor cells present. Excellent clinical outcomes have been documented with dose escalation to primary site (1). Similarly, to sterilize bulky pelvic nodal metastases, nodal dose escalation may be needed. There are other approaches to nodal dose escalation. Using EBRT boost, it is often difficult to escalate dose beyond 55 Gy because of the tolerance of the small bowel, the dose-limiting structure. To compensate error due patient motion or organ deformation, an effective dose escalating EBRT plan requires a setup margin. The nodal PTV increases OAR dose around the gross node. Even in a highly conformal EBRT boost plan, the SBD_1cc_ value can be similar to the prescription dose due to the 3 to 5-mm setup margin. We developed this CT guided HDR brachytherapy approach to dose escalate without going above standard small bowel tolerance. We were able to increase the nodal D_90%_ significantly higher than the small bowel dose.

The optimized tumor control dose to the positive pelvic nodes has not yet been determined, although NCCN guidelines (4) have stated that highly conformal boosts of an additional 10–15 Gy (approximately total dose, 55–60 Gy) may be considered for limited volumes of gross unresected adenopathy. Ariga et al. (20) reported that 52 of 57 patients (91%) with positive nodes (median maximum diameter, 15 mm; range 10–60 mm) were treated with 6–10 Gy EBRT boost (in 3–5 fractions; median total dose of EBRT for nodes, 56 Gy excluding cervical brachytherapy dose). Five of 57 node-positive patients (9%) developed pelvic node recurrences. Two of 52 boost patients (4%) experienced grade 3 small intestine late complications. Grigsby et al. (21) showed that 43 patients with CT lymph nodes >1 cm received a mean of 67.2 Gy for positive nodes combining EBRT dose and intracavitary cervical brachytherapy dose, and two (4%) had nodal recurrences. Although no clear dose response has been demonstrated, we believe there is evidence to support a need to improve local control of bulky node using dose escalation. Iheagwara et al. (22) suggested that a dose of ≥ 55 Gy in 25 fractions is adequate to control nodes < 2 cm in diameter in endometrial cancer patients. EMBRACE data (23) indicated a target nodal failure rate of 12% in patients treated with nodal boost, while 31% of node-positive patients underwent nodal surgery. It remains unclear whether bulky nodes can be sterilized using EBRT alone, without surgery. Our results showed dose escalation, for example to >60 Gy with brachytherapy may provide adequate control. The optimal dose to positive nodes for tumor control may be dependent on histology. This study included patients with squamous cell carcinoma, adenocarcinoma, or adeno-squamous cell carcinoma. It may be necessary to consider differences in tumor response according to tumor histology. Further investigation will be needed. Hacker et al. (24) noted that bulky nodes may require resection prior to radiation therapy in the belief that EBRT, although capable of sterilizing small node metastases, was unlikely to be successful against bulky nodal disease. However, it remains unclear whether debulking surgery is essential or possible for all patients with pelvic node metastases.

There are no established dose-volume histogram parameters, such as SBD_0.1cc_ and SBD_1cc_, for nodal brachytherapy. It has been reported that small/large bowel necrosis and obstruction necessitating surgery (grade 3 or higher late toxicity) are major side effects of dose escalation (9). The small bowel and sigmoid colon are dose-limiting OARs. The minimum dose in the maximum irradiated 2 cc of the small bowel may be relevant for telangiectasia. However, ulceration, necrosis, obstruction, and fistula are also important potential side effects in nodal ISBT. The dose at the D_0.1cc_ level may be related to the development of ulceration, necrosis, and fistula. Intestines such as sigmoid colon have inter-fraction and intra-fraction dose variation due to organ motion (25). When evaluating the DVH parameters to the bowel, the assumption of worse scenario may not adequately represent the reality. In this study, we used SBD_1cc_ as the bowel constraint.

One limitation of the present study may be that the success of ISBT is dependent on the technical skill of the brachytherapist. All patients in this study received nodal ISBT by a single-radiation oncologist (HK). Indeed, in one node (Lymph No.2 of the first case), the catheter was not placed perfectly in the center of the node, so the D_90%_ value of this node was lower than the SBD_1cc_ value (D_90%_: 54.7 Gyαβ10 vs. SBD_1cc_: 55.6 Gyαβ3, **Table 2**). However, this CT guided catheter placement technique is similar to many other CT guided procedure routinely performed by interventional radiologists. With adequate training we believe this procedure can be widely adapted by others. There is already another center that have demonstrated expertise and interest in this approach as Interventional Oncology Centers in Rome (26). The results in this study need to be validated in a larger multi-institutional study. Finally, the timing of ISBT may affect the dosimetric analysis. A dose coverage calculation depends on the size of the target. In patients with a primary tumor, the timing of nodal ISBT depended on primary site brachytherapy. Brachytherapy tends to be done after the node has responded to EBRT. In this study, the first nodal implant was performed either during the latter part of EBRT or about 1 week after EBRT. A similar approach with different brachytherapy timing may result in different outcomes. Another limitation is that the success of nodal ISBT may be dependent on the location of the positive nodes with respect to the blood vessel. Lymph nodes are often located close to the external/internal iliac vessels. It may be difficult to insert a catheter into nodes surrounded by vessels. In this study, implantation of nodal targets was based on the decision of a single radiation oncologist (HK). Based on his experience, larger nodes were easier to implant compared to smaller ones since it was easier to place the needle(s) in a larger target while avoiding the blood vessels. The nodal targets in this study were circular or oval-shaped; therefore, one or two needles per node were inserted into the central part of the node. Three or more needles per node might be needed for more irregularly shaped nodes. Another limitation of this study may be the nodal size limit of >15 mm, which was considered “bulky” by Grigsby et al. and Hacker et al. (21, 24). However, nodal ISBT may be more useful for sterilizing tumor cells in larger nodes, such as those >20 mm in diameter. Iheagwara et al. (22) reported that regional recurrence is significantly higher in patients with nodes ≥ 2 cm mm in diameter. The advantage of this technique may be limited to patients with larger nodes. In the future, artificial intelligence may provide the decision support with the application of nodal ISBT to improve clinical outcomes (27).

The number of patients in our study was small and the follow-up interval was short; thus, it is difficult to draw robust conclusions regarding late toxicity. Furthermore, it may be premature to evaluate small bowel late toxicity at after a median follow-up of only 26 months. Similarly, Yao et al. (5), Kishi et al. (6), and Yoshida et al. (7) showed the feasibility and safety of nodal ISBT, however, they also had small number of patients and had a shorter follow-up period. During HDR planning (see **Figure 3**), the lumbosacral plexus was not considered as an OAR because the dose constraint for that structure in ISBT combined with EBRT is unclear. Thus, lumbosacral plexus late toxicity should be assessed after a longer follow-up. Finally, Bacorro et al. reported the performance of primary site brachytherapy on pelvic nodes (28). In our study, we did not attempt to incorporate the dose from primary brachytherapy into the total nodal dose; further studies will be needed to do this if toxicity is observed.

## Conclusions

We described CT-guided HDR ISBT for bulky pelvic nodes and provided supporting evidence for the safety and efficacy of this approach. Such brachytherapy might offer a useful therapeutic option for dose escalation in unresected bulky pelvic nodes. Longer term follow-up is needed.

SBD0.1cc and SBD1cc = minimum dose in the maximum irradiated 0.1cc and 1cc of the

## Data Availability Statement

All datasets generated for this study are included in the article/supplementary material.

## Ethics Statement

The studies involving human participants were reviewed and approved by Juntendo University Hospital; approval no. 17-291. The patients/participants provided their written informed consent to participate in this study. Written informed consent was obtained from the individual(s), and minor(s)’ legal guardian/next of kin, for the publication of any potentially identifiable images or data included in this article.

## Author Contributions

HK and NY contributed conception and design of the study. HK and NY organized the database. HK performed the statistical analysis. HK wrote the first draft of the manuscript. I-CH, NY, SK, KN, YS, KF, YT, DO, RY, and KS wrote sections of the manuscript. All authors contributed to the article and approved the submitted version.

## Funding

This work was supported in part by JSPS KAKENHI grant number 16K19857 and 18K15562.

## Conflict of Interest

The authors declare that the research was conducted in the absence of any commercial or financial relationships that could be construed as a potential conflict of interest.

## Abbreviations

CT, computed tomography; CTCAE-4, Common Terminology Criteria for Adverse Events version 4.0; CTV, clinical target volume; D_90%_ and D_95%_, minimum dose delivered to 90% and 95% of the volume of the PTVn, respectively; EBRT, external beam radiotherapy; EF-IMRT, extended-field intensity-modulated radiotherapy; EQD2, equivalent dose in 2 Gy; GI, gastrointestinal; HDR, high-dose-rate; IPSA, inverse planning simulated annealing; ISBT, interstitial brachytherapy; JCOG-GCSG, Japan Clinical Oncology Group Gynecologic Cancer Study Group; MPR, multi-planar reconstruction; OAR, organ at risk; ^18^F-FDG-PET/CT, positron emission tomography with ^18^F-labeled fluoro-2-deoxyglucose/computed tomography; PRV, planning organ at risk volume; PTVn, nodal planning target volume; SBD_0.1cc_ and SBD_1cc_, minimum dose in the maximum irradiated 0.1 cc and 1 cc of the small bowel, respectively; SBRT, stereotactic body radiotherapy; TRUS, trans-rectal ultrasound; small bowel, respectively.
